# Patient Perceptions of Artificial Intelligence–Supported Shared Decision-Making in UK Primary Care for Multiple Long-Term Conditions: Qualitative Study

**DOI:** 10.2196/92518

**Published:** 2026-07-03

**Authors:** Charlotte Spurway, Sarah Flanagan, Jenny Cooper, Francesca L Crowe, Shamil Haroon, Tom Marshall, Leah Fitzsimmons, Eleanor Hathaway, Krishnarajah Nirantharakumar, Thomas Jackson, Sheila Greenfield, Louise Jackson

**Affiliations:** 1 Health Economics Unit, Department of Applied Health Sciences, College of Medicine and Health, University of Birmingham, Public Health Building, Edgbaston, Birmingham, B15 2TT, United Kingdom, 44 121 414 6486; 2 Department of Applied Health Sciences, College of Medicine and Health, University of Birmingham, Birmingham, United Kingdom; 3 Department of Metabolism and Systems Science, College of Medicine and Health, University of Birmingham, Birmingham, United Kingdom; 4 Department of Inflammation and Ageing, College of Medicine and Health, University of Birmingham, Birmingham, United Kingdom

**Keywords:** artificial intelligence, AI, multiple long-term conditions, qualitative research, primary care, shared decision-making

## Abstract

**Background:**

The prevalence of multiple long-term conditions (MLTCs) is increasing globally, leading to complex health care needs and polypharmacy. Shared decision-making (SDM) is important for supporting patient-centered care, yet barriers such as limited consultation time, discontinuity of care, and communication challenges hinder implementation. Artificial intelligence (AI) has the potential to support SDM by providing personalized, data-driven recommendations, particularly for medication management in patients with MLTCs.

**Objective:**

This study aimed to explore the perspectives of patients with MLTCs regarding SDM with their general practitioners (GPs) and to explore patients’ views about the use of an AI tool to support SDM, particularly in relation to prescribing decisions.

**Methods:**

This qualitative study explored the perspectives of 18 patients with MLTCs on SDM and the use of an AI tool prototype during GP consultations. Semistructured interviews used a simulated patient vignette and a visual AI tool dashboard to facilitate discussion. Participants were recruited through GP practices via the Clinical Practice Research Datalink and community-based organizations across the West Midlands. The data were then analyzed using thematic analysis.

**Results:**

Two overarching categories were identified: SDM in GP consultations and the AI tool for SDM. Within SDM, themes included communication and collaboration and system-level barriers, such as limited consultation time, lack of continuity, and fragmented records. Within the AI tool category, themes were related to practical design and implementation, implications for clinical practice and decision-making, and perceived risks and limitations. Participants valued the tool’s potential to summarize health information and support discussions but highlighted the need for clear explanations, accessible design, and clinician guidance. Concerns included time pressures, depersonalization, trust, and transparency, with participants emphasizing that AI should support rather than replace clinical judgment.

**Conclusions:**

Overall, patients perceived AI as a promising way to enhance SDM by improving communication and collaboration between patient and clinician. However, patients also had concerns about the accuracy and veracity of AI. The study provides recommendations for AI tools in GP consultations, emphasizing clear, accessible outputs and the use of lay language. AI tools should enhance rather than replace clinical judgment, be transparent about data sources, and be developed with diverse patient input to ensure inclusivity and usability, particularly for those with MLTCs.

## Introduction

### Background

The prevalence of multiple long-term conditions (MLTCs), defined as the coexistence of 2 or more chronic conditions in an individual, is rising globally due to aging populations and lifestyle factors [1,2]. MLTCs are associated with decreased quality of life, functional decline, and increased health care use, particularly as the number of coexisting conditions rises [1]. Managing MLTCs presents several challenges; for example, primary care practitioners must allocate limited resources to patients with complex needs, and prescribing guidelines focus on single conditions, often resulting in polypharmacy [3]. Polypharmacy, or the use of 5 or more medications daily [1], can lead to adverse events, inappropriate prescribing, and difficulties with adherence [4,5].

Shared decision-making (SDM) offers a way to address the complexity of MLTC management. SDM is a collaborative approach in which patients and clinicians make health care decisions together, incorporating patient preferences and the best available evidence [6,7]. Effective SDM relies on strong clinical communication skills, including rapport-building and structured consultations [6]. With MLTC, SDM is more complex, as clinicians must help patients prioritize concerns and make decisions within limited time and evidence [7].

Understanding how patients with MLTCs perceive and engage in SDM with a general practitioner (GP) is essential to improving care quality and outcomes. However, SDM is influenced by factors such as patient readiness, power dynamics, and limited organizational support [8]. A UK study found that both GPs and older patients with MLTCs expressed concerns about medicolegal vulnerability in SDM, arising from the risk of legal consequences if a negative outcome followed a shared decision [9].

Artificial intelligence (AI) offers the potential to support SDM and MLTC management. AI can perform tasks traditionally undertaken by health care professionals, including providing personalized information, decision aids, and predictive analytics to guide collaborative decision-making [10-12]. In GP consultations, AI tools could support medication decisions for patients with MLTCs by analyzing large datasets to generate tailored recommendations, optimizing treatment choices and minimizing the risks of polypharmacy. AI can consider individual patient characteristics and histories in ways beyond human capacity [13]. This is particularly valuable in the management of MLTCs, where adapting treatment plans to each patient becomes increasingly complex. As a result, AI could potentially be used to overcome the barriers of SDM for people with MLTCs by facilitating personalized discussions based on comprehensive data analysis and enhancing patient engagement through tailored information and decision aids.

### Objectives

For both patients and GPs, managing MLTCs can be complex, and although SDM is recommended, it is difficult to implement in practice due to polypharmacy, time pressures, and a lack of reliable evidence relevant to multimorbidity. AI could support personalized decision-making, but little is known about how patients perceive its use in GP consultations or how such tools should be designed and implemented to support patient-centered care. The Optimising Therapies, Disease Trajectories, and AI-Assisted Clinical Management for Patients Living with Complex Multimorbidity (OPTIMAL) project used 2 rounds of qualitative interviews to explore how AI could be used to analyze complex multimorbidity and prescribing data to support more personalized, safer decision-making for patients with polypharmacy [14]. This paper specifically explores the findings from a second round of interviews with patients who previously participated in interviews examining their perspectives on the potential benefits and risks associated with the use of AI in GP consultations [15]. In this study, the interviews focused on participants’ views relating to a prototype AI tool designed to support SDM. Participants reflected on how the tool could be used in practice and how it would support their own experiences of SDM and experiences of living with MLTCs. The following are the aims of the second round of interviews:

- Explore the perspectives of patients with MLTCs regarding SDM with their GPs
- Explore patients’ views on the use of an AI tool to support clinical decision-making to guide SDM, particularly in relation to prescribing decisions

## Methods

### Study Design and Setting

The data were collected through semistructured interviews conducted via telephone or video link (Zoom or Microsoft Teams) between January and February 2024. Patients were recruited from GP practices across the West Midlands, UK, with a total of 18 interviews conducted by 1 researcher (SF), who was a research fellow at the University of Birmingham at the time.

All participants had previously taken part in the first round of interviews exploring their perspectives on AI in health care consultations [15]. For the second round of interviews, a simulated patient vignette and a predictive algorithm prototype (AI tool) were developed by a multidisciplinary team. Vignettes may be used in social research to explore actions in context, clarify judgments, or provide a less personal way of discussing sensitive topics [16]. The simulated patient vignette was used to help participants explore and reflect on how the AI tool might function within a realistic GP consultation and helped convey complex concepts relating to AI-supported prescribing without requiring extensive explanation during the interview. The vignette was also developed and piloted with input from the participant advisory group (PAG).

The simulated patient vignette used in the study described a patient, “Janet,” with MLTCs to facilitate discussion (Multimedia Appendix 1). Alongside the vignette, the AI tool output provided participants with a visual representation of how the tool might function, using Janet’s case as an example (Multimedia Appendix 2). The AI tool was designed to support SDM during consultations between patients and GPs within a primary care setting by predicting risks for patients with MLTCs, considering their coexisting conditions and key metrics such as blood pressure and BMI. The tool could produce outputs comparing medication options by presenting the expected impact on the condition of interest alongside the predicted effects on other health conditions and key health metrics to support SDM.

The case study used data from the AI tool to support a decision about the most appropriate medication for Janet’s depression, considering her current health, medications, and health conditions. The AI tool presented her health metrics, conditions, and medications, alongside visualizations of the potential impact and outcomes of different antidepressants.

The interviews were guided by a topic guide, focusing on participants’ experiences with SDM and their feedback on the AI tool’s usefulness in supporting health care decisions (Multimedia Appendix 3). Participants were asked about their views on the AI tool, personal experiences of SDM in GP consultations, and how the tool might support SDM.

### Participants and Recruitment

For the first round of interviews, the study aimed to recruit up to 30 individuals with varied ages, genders, ethnicities, and numbers of long-term conditions. Recruitment continued until data saturation was reached, at which point additional data no longer generated new insights or themes [17]. Recruitment for the first round of interviews occurred between July and November 2023.

Eligible participants were recruited via the Clinical Practice Research Datalink (CPRD) Aurum database, an anonymized UK primary care dataset representative of the national population [18]. Using CPRD data on diagnoses, tests, prescriptions, and demographics, potential participants were identified and invited through the CPRD Patient Referral Service [19]. GP practices in the West Midlands were targeted to achieve a diverse sample, focusing on more deprived areas with higher proportions of patients from minoritized ethnic backgrounds and with MLTCs. CPRD contacted participating GP practices, which reviewed lists of eligible patients and invited them to take part.

Participants were eligible if they were aged 18 years or older who have not opted out of contributing data to the CPRD database. Participants were also required to have self-reported MLTCs, defined as the coexistence of 2 or more chronic conditions. Participants could also be a carer for someone with 2 or more conditions; however, no carers were recruited for this study. Exclusion criteria included having a terminal illness or lacking the capacity to provide informed consent. Those interested in taking part contacted the research team directly to ask questions, provide consent, and arrange an interview.

Additional recruitment was conducted via community-based methods, including flyers distributed through voluntary and community organizations such as patient support groups. Interested individuals were sent a participant information sheet and offered the opportunity to discuss the study with a member of the research team before deciding whether to participate.

Of those who participated in the first round of interviews, individuals were asked whether they consented to be contacted for a second interview. Twenty-eight participants gave consent and were subsequently contacted by email. The email invitation outlined the aims of the second interviews and what participation would involve. Of those who initially agreed to be contacted, 10 declined or were unavailable. In total, 18 participants completed a second interview.

### Interviews

For the second round of interviews, participants were sent the simulated patient vignette, “Janet,” and a PDF of the AI tool dashboard in advance, either by email or by post. They were asked to review these documents prior to the interview. At the start of each session, participants were asked if they had reviewed the materials and whether they had any questions. The interviewer (SF), who also conducted the first round of interviews, clarified any aspects of the vignette or AI tool for participants who found them challenging to understand. Interviews lasted up to 60 minutes. While the sample size for the follow-up interviews was guided by the number of participants who agreed to take part in the second interview, analysis indicated that no substantially new themes were emerging across interviews, suggesting sufficient thematic depth and consistency within the dataset [20].

### Patient and Public Involvement

People with lived experience of MLTCs were involved throughout the OPTIMAL study. A PAG of 9 members, including individuals with direct or carer experience of 4 or more long-term conditions, was established at the study’s inception. Recruitment aimed to ensure diversity in age, sex, ethnicity, and condition type. The PAG contributed to developing the topic guide, reviewing participant-facing documents, providing feedback on emerging analyses, and reviewing findings.

### Data Analysis

Interviews were audio-recorded and transcribed verbatim by a transcription company contracted by the University of Birmingham. Personal identifying information was removed from the transcripts prior to analysis. The transcripts were analyzed using the thematic analysis methods outlined by Braun and Clarke [21]. Following thorough familiarization with the transcripts, the researchers assigned codes to the data using an inductive approach. The codes were then organized by grouping related codes into overarching categories relating to SDM in GP consultations and the AI tool. Initial line-by-line coding generated a large number of descriptive codes capturing participants’ views on communication, trust, time pressures, and perceptions of AI. Codes were iteratively compared and refined, and related codes were grouped into higher-order categories. These categories were then developed into themes that captured patterns across the dataset.

The coding and analysis of the data were undertaken by 2 researchers (SF and CS). As the codes and themes were identified, these were discussed with the PAG and the multidisciplinary research team to identify further areas for exploration. Microsoft Word and Excel (Microsoft 365) were used to read the transcripts and support coding and theme development [22].

The COREQ (Consolidated Criteria for Reporting Qualitative Research) checklist was completed to support transparent reporting. The checklist is provided in Checklist 1, and the corresponding reflexivity statement is provided in Multimedia Appendix 4.

### Ethical Considerations

Electronically completed written consent or audio informed consent was collected from participants. Ethical approval for the study was obtained from the National Health Service Research Ethics Committees (reference: 22/SC/0210). Ethical approval to use the anonymized data from the CPRD dataset to select eligible participants was obtained from the CPRD Expert Review Committee and the Central Advisory Committee (reference: 21_000683). All interviews were transcribed, anonymized, and stored securely in accordance with UK data protection regulations and the University of Birmingham security policies. Participants received a £15 (equivalent to US $20.12 as of June 17, 2026) voucher as a thank you for their time.

## Results

### Participant Characteristics

**Figure 1.**
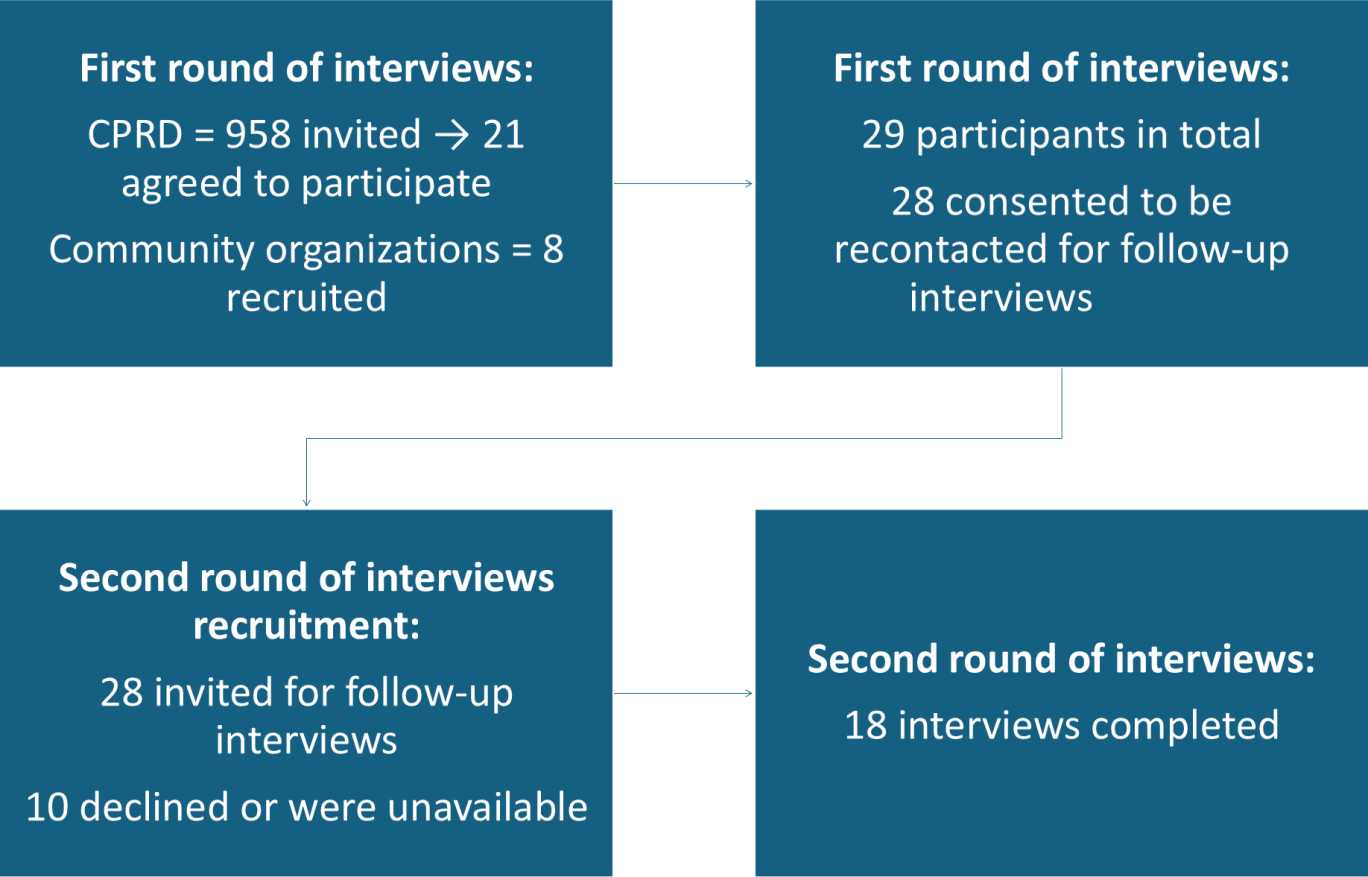
Recruitment flow diagram for the first and second rounds of interviews. CPRD: Clinical Practice Research Datalink.

A total of 18 interviews were conducted. Figure 1 presents a flow diagram of recruitment for the interviews. Among the participants who completed the second interview, 9 were female and 9 were male. The majority were White British (n=14, 77.8%), with 3 identifying as Asian or British Asian and 1 as mixed ethnicity. Age distribution was as follows: 6 participants aged 70 to 79 years, 3 aged older than 80 years, 5 aged 60 to 69 years, 3 aged 50 to 59 years, and 1 aged 40 to 49 years (Table 1). In the quotations presented, each participant is identified by their ID number, sex (female [F] or male [M]), and age.

Two overarching categories were used to organize the themes and subthemes: SDM in GP consultations and the AI tool for SDM in GP consultations. Within the SDM category, 2 themes were identified: the role of communication and collaboration and system-level barriers associated with SDM. Within the AI tool category, themes included practical considerations for AI tool design and implementation, implications for clinical practice and decision-making, and perceived risks and limitations of the AI tool.

**Table 1.** Participant characteristics (N=18).

Characteristics		Participants, n (%)
Sex		
Female		9 (50)
Male		9 (50)
Age range (y)		
40‐49		1 (5.56)
50‐59		3 (16.67)
60‐69		5 (27.78)
70‐79		6 (33.33)
>80		3 (16.67)
Ethnicity		
White British		14 (77.78)
Asian or British Asian		3 (16.67)
White and Black African		1 (5.56)
Number of long-term conditions		
2‐3		6 (33.33)
>4		12 (66.67)

### SDM in GP Consultations

The interviews identified several circumstances within GP consultations that support or hinder SDM for patients with MLTCs.

#### Communication and Collaboration for SDM

The role of communication and collaboration was important to participants in enabling SDM. Several participants reflected on their experiences, particularly highlighting times when communication was poor. Open and 2-way GP-patient communication was considered essential for SDM. A lack of communication led participants to feel uninvolved in their care and created a lack of transparency about their conditions and treatments. As 1 participant explained:

*Because we get told we’ve got to take this medication and this medication and we’re not absolutely sure what it’s doing either. It would be good if it was explained to us.*

[ID14, F, 79]

In addition to communication, collaboration between GPs and patients was identified as a key element of SDM. This collaborative approach allows patients to actively participate in managing their conditions, with effective communication serving as a necessary foundation for these discussions. The majority of participants expressed a desire to be involved in decisions about their care, although some reported being happy to rely more on doctor-led decision-making, particularly when the condition was perceived as less serious:

*If it’s for, I don’t know, if it’s for a rash or something like that, then I’m completely relying on the doctor to come up with the answer … If it was something far more serious like whether to have a knee replacement then naturally I would want to consider all the implications of a serious operation like that.*

[ID4, M, 68]

The interviews showed that the majority of participants wanted SDM in practice. However, some participants felt that decisions were imposed on them rather than reached collaboratively.

*It’s them telling you, in most cases. Not always, but most cases I think … And they just sort of brush it off like it’s you know, “Get on with it” which isn’t very helpful.*

[ID6, F, 72]

Participants reported feeling more engaged in SDM when treatment options and alternatives were clearly explained, and when they were actively encouraged to share their opinions and question recommendations.

#### System-Level Barriers to SDM

Several barriers can affect the ability of GPs and patients to engage in SDM. Participants identified that the limited time available during GP appointments was a barrier to SDM. Some questioned whether the standard 10-minute consultation allows sufficient time to reach a shared decision. A lack of continuity of care led patients to see multiple GPs and repeatedly recount their medical history, reducing time for meaningful discussions.

*Well you used to be able to [see the same GP] but you can’t any longer. […] so you’ve got to go through all the rigmarole of telling them why you’re there and what the background is before and then your ten minutes are up before you know.*

[ID17, M, 76]

Some participants noted that seeing different GPs could provide fresh perspectives on their conditions and medications. SDM experiences ranged from exclusion to active involvement, influenced by clinician comfort, the clinician-patient relationship, and patient confidence in questioning decisions, as one participant explained:

*But also it depends which doctor I see as well. Some are more understanding than others and they have a bit more empathy with you and they will sit and talk. Where others you’ll go in and it’s like “OK, this is a prescription.”*

[ID23, F, 53]

For patients with MLTCs, a lack of integrated IT systems and shared clinical records has been identified as a major barrier, requiring patients to repeatedly explain their history, leading to frustration and missed opportunities for informed discussions.

### AI Tool for SDM in General Practice Consultations

For the second part of the interview, participants were given the vignette and the AI tool output. They reflected on its usability, suggested improvements, and considered its role in SDM.

#### Practical Considerations for AI Tool Design and Implementation

##### Usability and Value

Many participants found the tool useful and wanted access to it throughout GP consultations. Participants reported the AI tool was helpful when it showed a summary of their health indicators, which may provide an incentive for behavior change.

*Because if you have something like this and you—oh let me go and have a look at that again … Or maybe if I reduce this, this will get better so let me—I would think that would be, yeah, might be an incentive or motivation to people to improve their health you know.*

[ID15, F, 57]

The understanding of the AI tool, however, was not straightforward for everyone. Participants emphasized the need for doctors to explain the tool and clarify the source of its data. Some also suggested simplifying the content to make it easier to interpret, helping patients feel confident to ask questions. Participants reported mixed experiences, with clarity varying and some struggling with acronyms and medical terminology. As 1 participant explained:

*What is EGFR? HbA*_*1c*_ *? And issues like units of drinking, you know, and what does “unit” mean? It needs to be simplified.*

[ID5, M, 71]

##### Presentation and Accessibility

Visual features, such as graphs and diagrams, were generally appreciated for simplifying complex information. Participants suggested minor design improvements, including adjusting typeface size and ensuring color schemes were accessible, especially for people with visual impairments or color blindness.

*Also not sure if the colours would be accessible … I struggle when I look at it to see the blue and the white. The white on the blue is very difficult for me to—there are people who are colour blind.*

[ID15, F, 57]

Not everyone favored the visual presentation of information. Some participants said they would prefer verbal explanations, while others suggested that those unfamiliar with graphical formats might find the outputs harder to interpret:

*If you’re not used to graphical, visual presentation of data, you might struggle.*

[ID19, M, 64]

Participants noted that some individuals, such as those with lower health literacy, older adults, and people with language barriers or limited English proficiency, might struggle to understand the outputs. Participants expressed concern that non-English speakers might not benefit, thereby limiting the tool’s usefulness for SDM:

*And what if your first language isn’t English you know. […] are they going to be, you know it’s not going to be sort of like translated or whatever in their language.*

[ID23, F, 53]

### Implications for Clinical Practice and Decision-Making

#### Time and Resources

Many participants queried whether GPs had enough time to review the AI tool during consultations, particularly as time is already limited.

*What happens now, you go in and you’re allowed five or ten minutes the most. Is that enough time to go through everything?*

[ID8, F, 83]

Several participants identified limited time as a potential barrier to introducing the AI tool to enhance SDM. Some suggested allowing longer appointment times when the AI tool is used or providing the tool to patients in advance:

*The appointment could be longer, especially as I say, if this person, me or whoever, has got to take four different medications. You’d want to know how beneficial it is going to be to you.*

[ID14, F, 79]

For 1 participant, they felt that GPs already use on-screen information to make quick decisions, as they explained:

*I’ve got the impression that this information is on their screens anyway. And they scan. So they get some recommendations and then they make a decision themselves …*

[ID19, M, 64]

#### Impact on Clinician Decision-Making

The interviews also found differing participant opinions around the impact of the AI tool on clinician decision-making. Some participants worried that increased reliance on the tool might reduce doctors’ use of their own knowledge, experience, and judgment, potentially making them feel pressured to follow the AI tool’s recommendations, even if they do not fully agree:

*[…] The third one I think, is the doctor under pressure potentially I think to conform to the given algorithm?*

[ID5, M, 71]

However, some participants thought the tool would be useful in guiding doctors’ decision-making, acting as a prompt to support decisions or trigger medication reviews:

*If he’s missed something, when you go in for something and thought “Oh, I’d forgot about his kidney. Oh we’ll have to think about that” or “Oh it’s nice to see the blood pressure here. It’s nice to see this, this, this.” That’s a prompt for them as well.*

[ID13, F, 65]

Therefore, some felt that it would be useful as a prompt for clinicians, helping to highlight relevant clinical information and support SDM, while others also emphasized that it should not replace clinical judgment.

### Perceived Risks and Limitations of the AI Tool

#### Depersonalization

Some participants believed the AI tool could enhance communication between GPs and patients, encouraging SDM. Rather than the doctor making a quick decision, for example saying, “Oh, that’s that. Give her a script” (ID13, F, 65), participants felt the tool could support both parties in exploring options together. Some were concerned that AI might reduce human interaction and empathy in consultations. Others felt a generic AI approach would not suit everyone, particularly if patients were not fully honest about health behaviors such as alcohol consumption. There were also concerns that it might encourage a standardized model of care that overlooks individual circumstances, including broader social, physical, and psychological contexts.

*The failure to look at the patient as an individual and taking account of the individual’s social, physical or psychological context because you’re dealing with a particular algorithm.*

[ID5, M, 71]

These concerns reflect apprehension about standardization and whether algorithms can encapsulate individuals’ lived experiences and, hence, be informative for another individual.

#### Trust and Transparency

Participants also raised concerns about the introduction of an AI tool to support SDM around trust and transparency of the tool. Some were curious about how the tool worked, others questioned the accuracy and trustworthiness of the data:

*I’d be very interested in finding out more about the sources or the statistics were up to date … old data has proved to be inaccurate or incorrect.*

[ID4, M, 68]

Participants also questioned the trustworthiness of AI around data sources and potential pharmaceutical industry influence. They emphasized the clinician’s role as an intermediary, suggesting that trust depends on doctors explaining and contextualizing AI outputs rather than patients accepting them at face value. It also reflects uncertainty about the transparency of the tool’s predictive data and concerns about how the “authority” of the AI tool may impact the patient and clinician’s agency in decision-making. For 1 participant, they also wondered whether the AI tool was aimed to improve patient care or simply cut costs:

*If AI gets developed or comes in, it could save the NHS millions. I mean is that what it’s all about? Is it all about saving money or getting a better, improvement for the patient?*

[ID26, M, 76]

## Discussion

### Principal Findings

This study explored how an AI tool could be used in GP consultations to support SDM for people living with MLTCs. Participants engaged with the AI concept while reflecting on the complexities of managing their MLTCs, unfamiliar terminology, and receiving conflicting health information. Although many participants valued involvement in decisions about their care, experiences were shaped by continuity with the same GP, consultation time constraints, and communication quality.

These findings echo broader evidence that SDM depends not only on patient preference but also on systemic conditions that enable meaningful dialog [6,23]. While patients are generally supportive of SDM, their engagement is influenced by factors such as knowledge, continuity of care, and time [8]. This highlights the systemic, cultural, and interpersonal barriers that prevent patients’ full participation in the decision-making process [24].

Although grounded in their own experiences of managing MLTCs, participants often reflected on how the AI tool might affect others, particularly those with lower health literacy, language barriers, or accessibility needs. Participants questioned whether AI-driven recommendations might alleviate or exacerbate these existing challenges. Concerns were also raised around whether AI-driven recommendations would make health care less personalized, and some questioned the trustworthiness of the data used in the AI tool. Participants were concerned that AI tools could reconfigure the dynamics of the decision-making process away from their own priorities and toward external pressures, such as cost-saving measures.

This study is 1 of the first to explore how an AI tool can support SDM in GP consultations for people with MLTCs in the United Kingdom. There is a particular lack of evidence on AI-supported SDM within the structure of UK primary care, where consultation length, continuity of care, and challenges associated with MLTCs differ from other health systems. This study therefore offers novel, context-specific evidence. Understanding patients’ perspectives can inform the development of AI tools that are acceptable and appropriate, particularly for those with high treatment burdens [7]. Such patients typically attend more frequent appointments and are prescribed multiple medications compared to those without MLTCs, which can impact adherence and engagement with care [7,25].

While SDM is well explored, the use of AI to support it in MLTCs remains underdeveloped. Current guidelines are based largely on single-disease models, as clinical trials often exclude people with multimorbidity, increasing the risk of polypharmacy and complications [7,26]. While existing studies provide general support for the use of AI in primary care settings [27,28], there is limited evidence on how AI can be applied specifically to facilitate SDM within GP consultations for patients with MLTCs.

In this study, the AI tool was received positively by participants, although some worried it could complicate consultations, which already have limited time. In addition, some participants feared the tool might be applied in a “one-size-fits-all” manner, removing personalization from consultations. For participants, this depersonalization meant that the AI tool would not be able to consider the patient’s broader social, physical, or psychological context in the way that doctors can. These concerns reflect worries about how AI may standardize care for individuals, without considering how MLTCs and wider individual circumstances shape care needs.

These concerns are echoed in a study of primary care physicians in Germany, which found that AI frequently gave irrelevant recommendations and generated patient alerts that were not clinically appropriate [29]. This reinforces the importance of clinical oversight of AI tools. Participants in this study felt that AI could not completely replace GP decision-making and emphasized the importance of doctors continuing to use their own knowledge, experience, and judgment. Participants also noted that AI outputs could influence clinician decision-making, potentially shifting power dynamics in consultations if doctors feel pressured to follow algorithmic guidance, highlighting the need to balance AI support with clinician and patient autonomy.

These findings align with research from other studies, emphasizing the importance of physician oversight in AI-supported care [11]. Similarly, a recent study with health care professionals found that while AI tools hold promise for the management of clinical complexity, they must also provide clear rationale for recommendations, preserve clinician autonomy, and integrate within existing workflows [30]. Overall, AI offers promising opportunities to support SDM, but challenges remain regarding usability, patient understanding, and preserving human elements in care. Balancing technological innovation with patient-centered values is important to ensure tools enhance, rather than replace, clinical relationships.

### Strengths and Limitations

This qualitative study has some strengths and limitations. A comprehensive recruitment approach was adopted, incorporating invitations via the CPRD database and engagement with community, patient, and third-sector organizations. This approach supported the inclusion of participants with diverse experiences and characteristics, enhancing the transferability of the findings [31]. Guided by a multidisciplinary research team, the study applied robust methods for data collection and analysis. Semistructured interviews enabled in-depth exploration of patients’ experiences with MLTCs, SDM, and AI, providing insight into social, cultural, and personal factors shaping views. Furthermore, the use of the COREQ checklist provides a clear and transparent account of its analytic approach, enabling readers to understand how interpretations were developed.

The lower-than-expected response rate may have been influenced by the time commitment required for participation, involving two 60-minute interviews, highlighting the need to consider participant burden in future studies. Additionally, as noted elsewhere, individuals without direct experience of AI may find it difficult to conceptualize or engage with such topics, particularly when examples of AI applications are not clearly defined [32]. Participants who decided to take part in the study and attended both the first and second interviews may have had a particular interest in the topic or been disproportionately more digitally literate or more physically well, meaning the sample might not fully represent the broader population of patients with MLTCs.

In addition, while the sample included some diversity, individuals from ethnic minority backgrounds were underrepresented. As a result, certain perspectives may not be fully captured in this analysis, which is particularly relevant given the potential for bias in AI systems [33].

In this study, 1 of the central limitations is related to the fact that the study used a static AI prototype, presented as a PDF, which served only as a visual guide and was not interactive. This means responses reflected expectations rather than real-world use. The early prototype offers some benefits, including obtaining direct feedback about preferences and requirements at an early stage, supporting responsible AI development. However, the findings are speculative rather than based on genuine user experience. Therefore, the design-related conclusions drawn from this study need to be tested, and further research is required to determine whether a real-world AI tool for SDM and MLTC would produce the same findings as those presented here.

### Implications for Practice, Policy, and Research

Despite its limitations, this study offers recommendations for the design of AI tools for use during GP consultations. These recommendations are separated into 3 design areas. The first relates to AI tool design. AI tools that produce outputs for patients need to present a mixture of text and visual information in a clear and simple manner. The design also needs to consider accessibility issues that can affect readability, such as color choices for color blindness and how outputs can be made accessible to people with visual impairments.

The second area relates to how the AI tool is communicated. The terminology used needs to be in lay language, and health care professionals using the tool to support SDM need to be able to explain it adequately to patients. Equality, diversity, and inclusion should also be considered in the design, including involving patient and public groups and providing scope to produce outputs in languages other than English. Future research should include diverse populations, including minority ethnic groups, people with language barriers, and those with sensory or learning disabilities, to ensure AI tools are inclusive and do not risk exacerbating existing inequalities [34].

The third area relates to how AI tools can be used in practice. Doctors need adequate time to explain the tool, and tools should be designed to enhance SDM rather than replace clinical decision-making. Patients in this study questioned data sources and transparency, so it is important that data provenance is communicated clearly. Furthermore, patients require support to understand AI outputs, and GPs need sufficient time and training to integrate these tools without compromising personalized care.

### Conclusions

This study demonstrates how AI could be used to support SDM in primary care consultations for patients living with multimorbidity. Participants largely supported the idea of using an AI tool to improve communication and collaboration with their GPs, recognizing its potential to present complex information in a more accessible way and to prompt more meaningful dialogue during consultations. However, they also highlighted the importance of clear explanations and the need for the tool to complement rather than replace human clinical judgment. Future research should focus on evaluating how such tools are implemented and used in real-world primary care settings, including their impact on consultation dynamics, patient understanding, and clinician workload over time. Further work is also needed to explore longer-term patient experiences and to assess acceptability and effectiveness across more diverse patient groups to inform equitable implementation.

## Supplementary material

10.2196/92518 Multimedia Appendix 1 Simulated patient vignette.

10.2196/92518 Multimedia Appendix 2 Artificial intelligence tool prototype.

10.2196/92518 Multimedia Appendix 3 Interview topic guide.

10.2196/92518 Multimedia Appendix 4 Reflexivity statement.

10.2196/92518 Checklist 1 COREQ checklist.

## Abbreviations

AI: artificial intelligence
COREQ: Consolidated Criteria for Reporting Qualitative Research
CPRD: clinical practice research datalink
GP: general practitioner
MLTC: multiple long-term condition
OPTIMAL: Optimising Therapies, Disease Trajectories, and Artificial Intelligence-Assisted Clinical Management for Patients Living With Complex Multimorbidity
PAG: patient advisory group
SDM: shared decision-making
