# Supplementary material for: Patient Perceptions of Artificial Intelligence–Supported Shared Decision-Making in UK Primary Care for Multiple Long-Term Conditions: Qualitative Study
Source: J Med Internet Res. 2026 Jul 3;28:e92518. doi: 10.2196/92518 (PMC13331396; doi:10.2196/92518)
Supplement: Multimedia Appendix 1 [file jmir-v28-e92518-s001.docx]

**Patient summary**

**Background**

Janet is a 72 year old retired secretary who lives on her own. She had a cardiac event called Myocardial infarction (MI) three years ago.

She has high blood pressure (hypertension) diagnosed over 10 years ago, type 2 diabetes diagnosed 6 years ago, and she has chronic kidney disease stage 3a.

Her diabetes is stable and relatively well controlled and her kidney function is also stable.

Her blood pressure is 134/82 – stage 1 high blood pressure.

Janet is a non-smoker and drinks occasionally. She does minimal exercise.

Her medications are:

Ramipril 10mg - ischaemic heart disease, Chronic Kidney Disease, diabetes, hypertension

Amlodipine -5mg hypertension

Glyceryl trinitrate spray - as required. - ischaemic heart disease.

Aspirin 75mg - ischaemic heart disease

Bisoprolol 10mg - ischaemic heart disease, hypertension

Atorvastatin 80 mg - ischaemic heart disease

Metformin - modified release 500mg BD - diabetes.

**Today’s consultation:**

During today’s consultation, Janet shares that over the last 6 months, she had been struggling with sadness (her husband dies 10 years ago), poor sleep, reduced appetite and interest in activities she used to enjoy.

She feels quite "empty" and tired a lot of the time. She often thinks it might be better if she did not wake up the next day, but she does not have any plans for suicide. **The GP has diagnosed her with moderate to severe depression and she is now asking whether she could try some medication.**  She knows her son has been taking fluoxetine for many years for his depression and he found this really helpful.  She is also willing to try some CBT.

The GP enters Janet’s current medication, history and current health indicators into the algorithm (AI tool) to show:

1. The predicted impact upon depression after one year - comparing having no medication with taking an anti-depressant (in this case fluoxetine as Janet has heard positive things about this from her son).
2. The potential impact after one year of taking fluoxetine compared with four other anti-depressants upon one of her health indicators – in this case – weight.
3. Finally, the output will show the 1 year risk of anti-depressant medication on her other medical conditions e.g. no change, worsen, or improve.

Please note, in the consultation, the AI output (Dashboard) included will be shown to the patient on a computer screen, rather than on a paper copy
