# Supplementary material for: Patient Perceptions of Artificial Intelligence–Supported Shared Decision-Making in UK Primary Care for Multiple Long-Term Conditions: Qualitative Study
Source: J Med Internet Res. 2026 Jul 3;28:e92518. doi: 10.2196/92518 (PMC13331396; doi:10.2196/92518)
Supplement: Multimedia Appendix 2 [file jmir-v28-e92518-s002.pdf]

Dashboard

Patient: **Janet Dawn**

Patient Information

Age: 72 years

Gender: Female

Ethnicity: White

Medical Conditions:

- Hypertension
- Type 2 Diabetes
- Chronic Kidney Disease Stage 3a

Health Indicators

BMI	Blood Pressure	eGFR	Hba1c	Smoking	Drinking
**32**	**134/82**	**55**	**52**	**No**	**2.1** units

List of medication the you are currently on:

- Ramipril **10mg**
- Amlodipine **5mg**
- Aspirin **75mg**
- Bisoprolol **10mg**
- Atorvastatin **80mg**
- Metformin **1mg**
- Glyceryl Trinitrate spray

1-year outcome for 10 people like you with depression

Medication: Fluoxetine

No Medication:

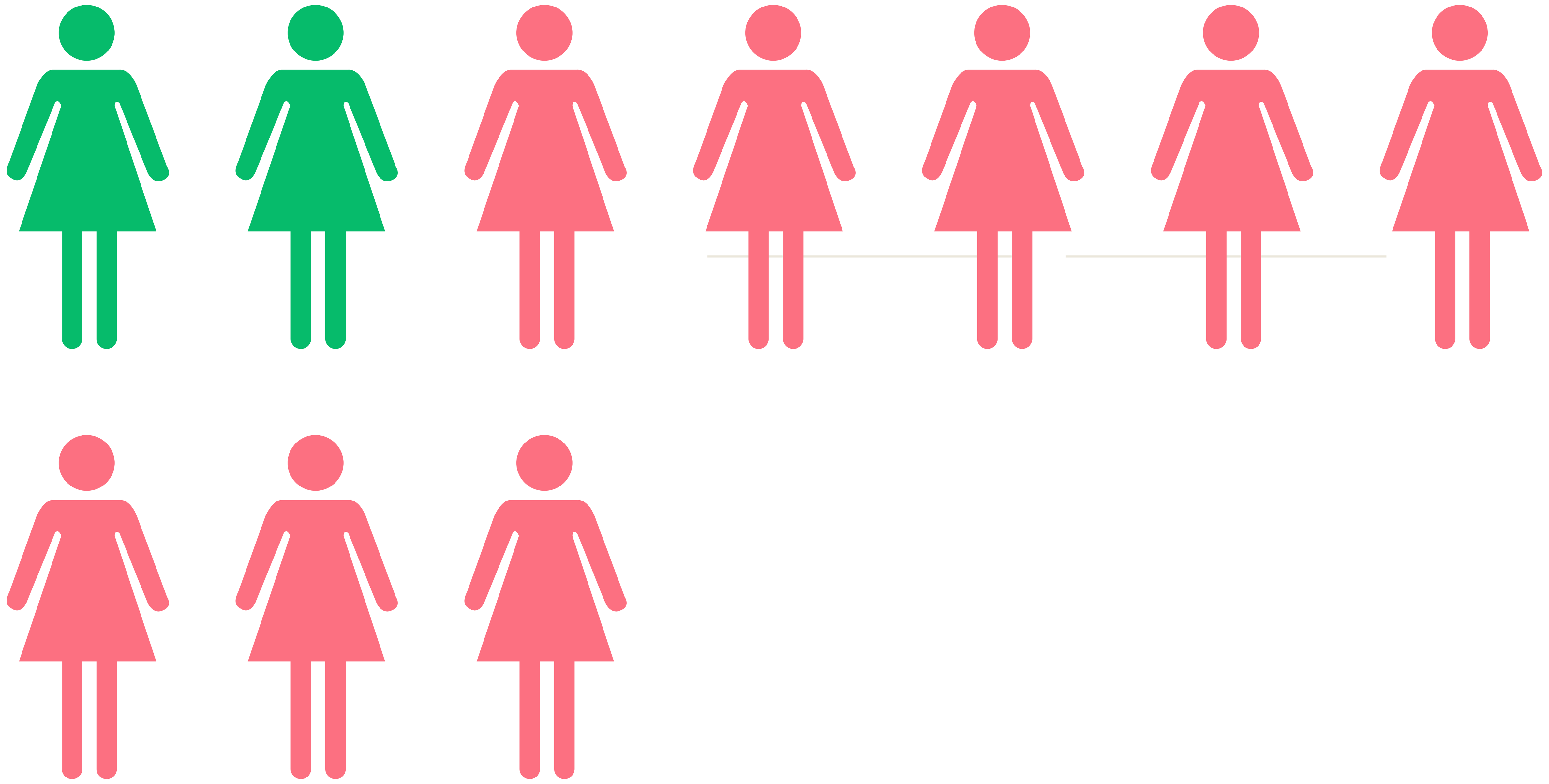

2 out of 10 people show a reduction in symptoms.

On Fluoxetine:

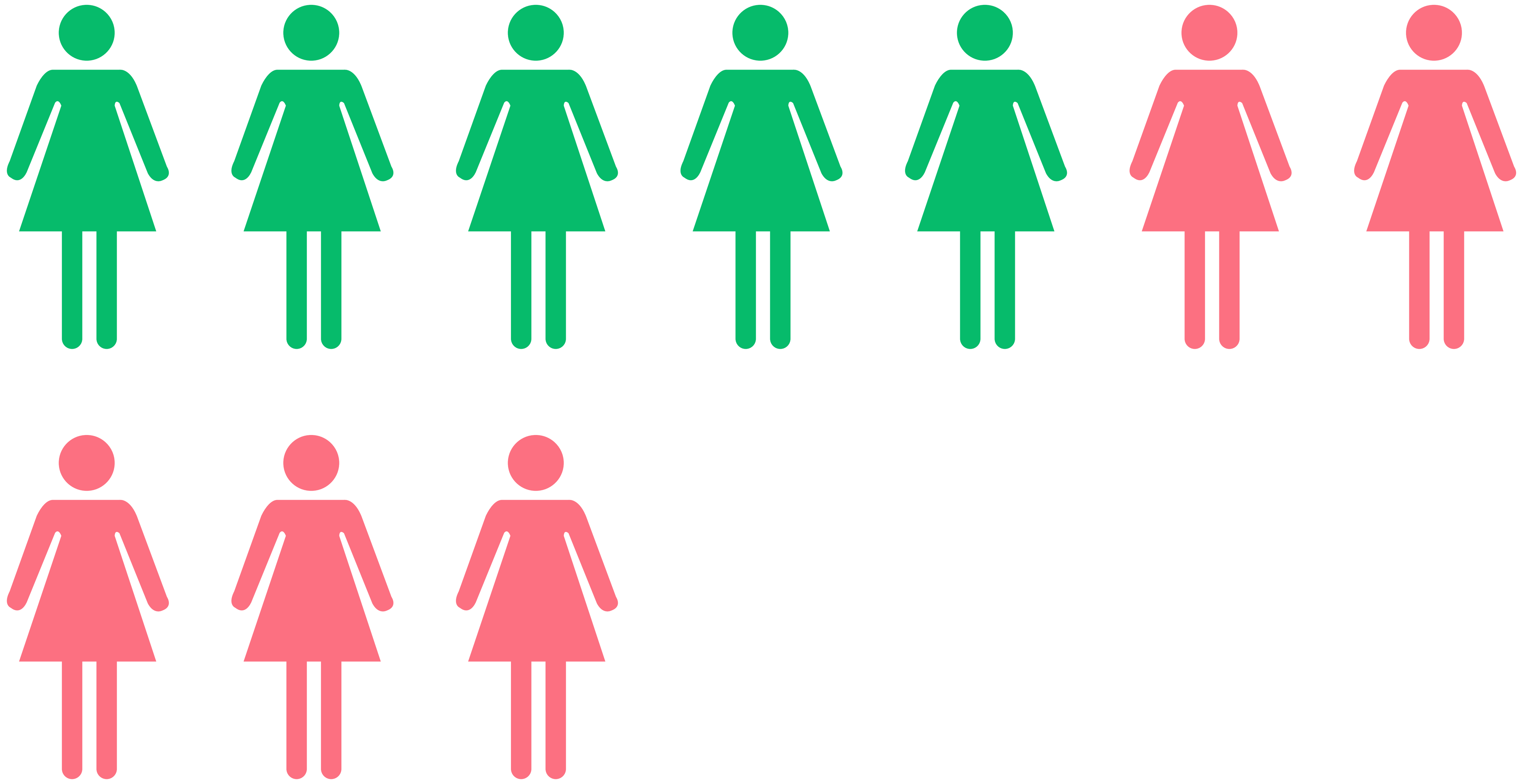

5 out of 10 people show a reduction in symptoms.

Your 1-year Health Indicators:

Indicator: Weight

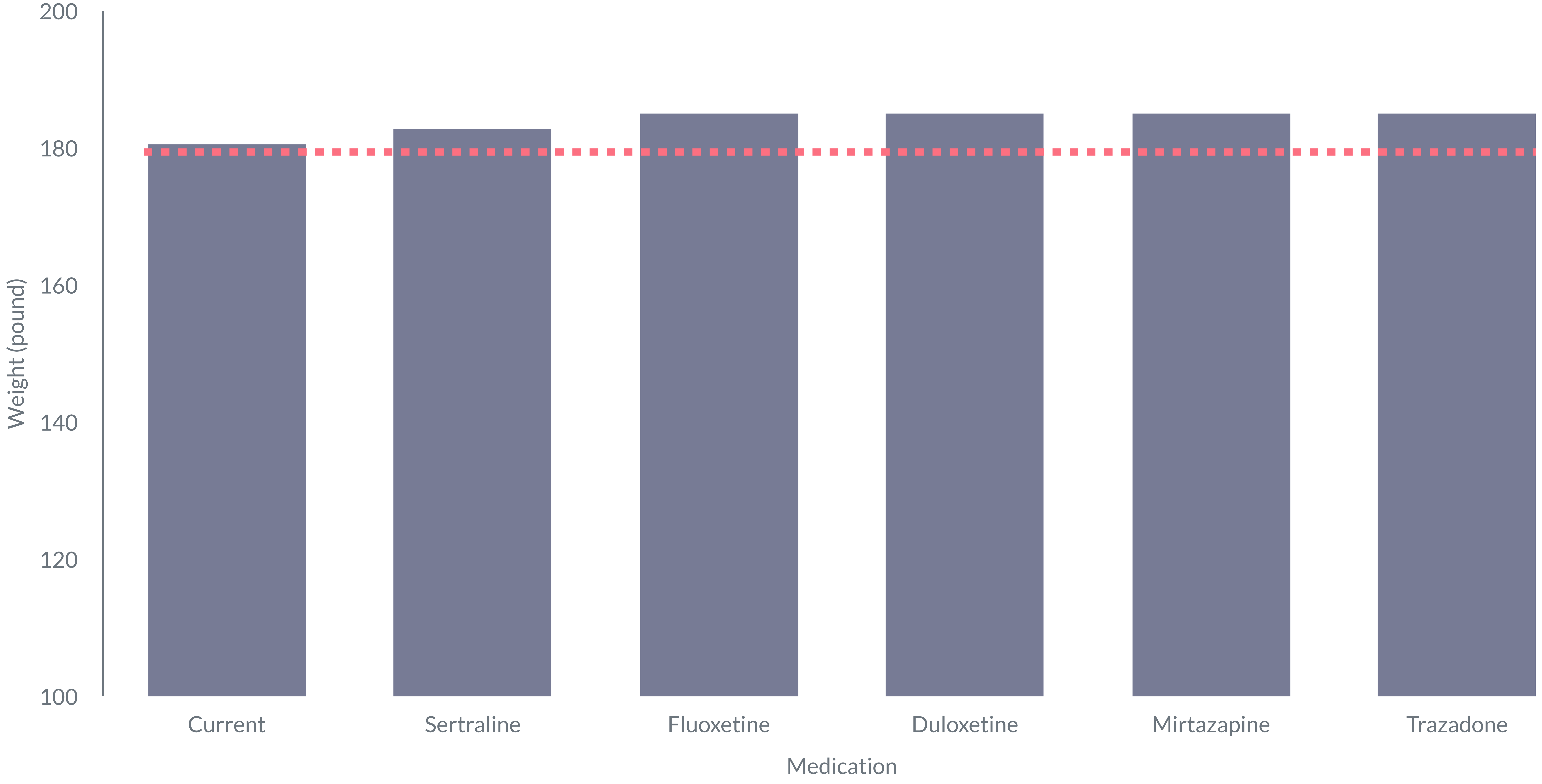

Your 1-year risk of anti-depressant on current medical condition:

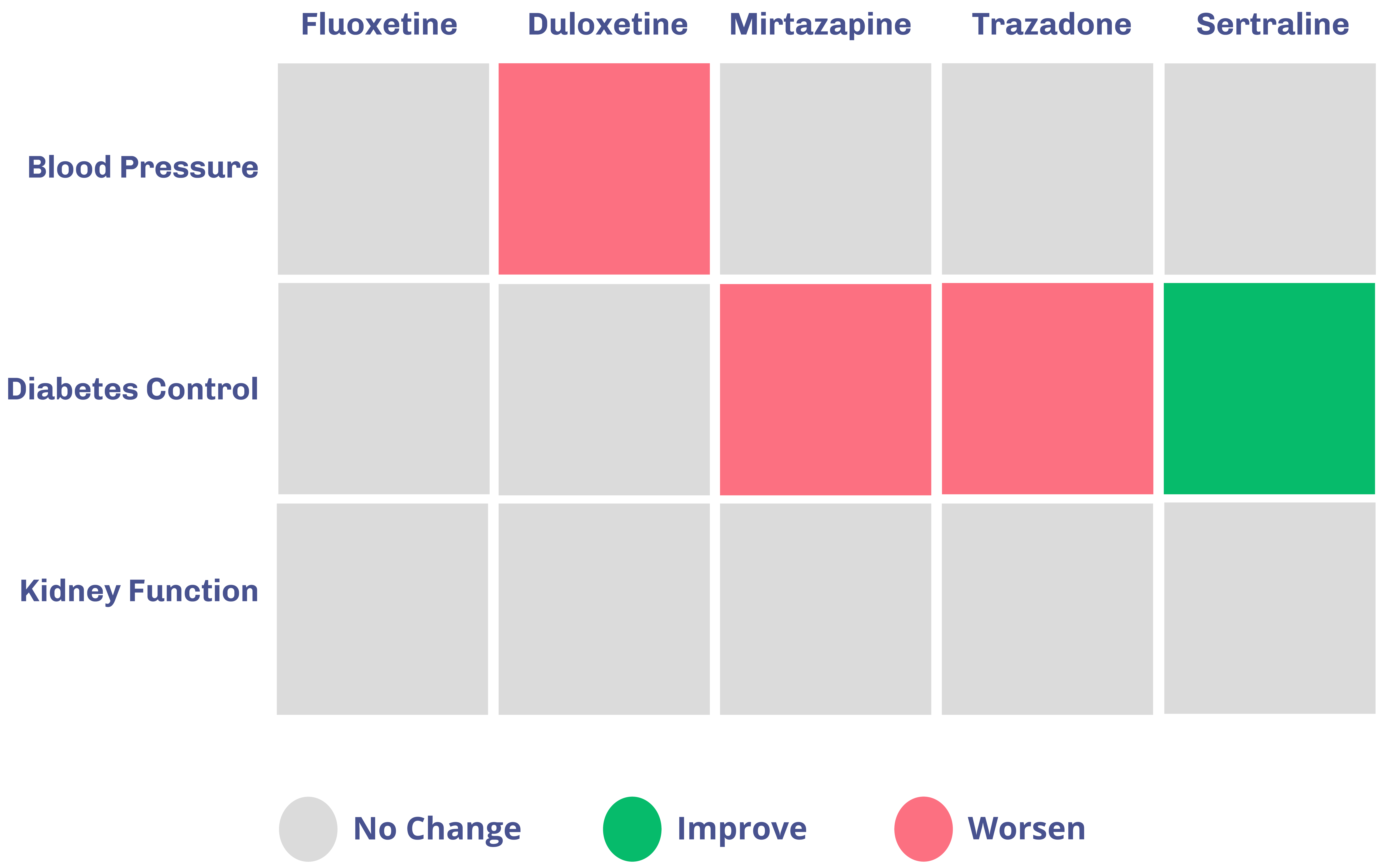

© **2023 OPTIMAL Project.** All rights reserved. Data are presented for illustration purposes only.
