# Supplementary material for: Patient Perceptions of Artificial Intelligence–Supported Shared Decision-Making in UK Primary Care for Multiple Long-Term Conditions: Qualitative Study
Source: J Med Internet Res. 2026 Jul 3;28:e92518. doi: 10.2196/92518 (PMC13331396; doi:10.2196/92518)
Supplement: Multimedia Appendix 3 [file jmir-v28-e92518-s003.docx]

**OPTIMIsing therapies, disease trajectories, and AI assisted clinical management for patients Living with complex multimorbidity**

**(The OPTIMAL study):**

**Interview guide: Patient Interview**

**This is the starting topic guide. The overarching objectives will remain the same, but questions and prompts will be developed as interviews are undertaken to incorporate any important themes emerging from the interviews.**

**Before the interview begins**

- *Ensure the participant has read the information leaflet*
- *Ensure the participant has had the opportunity to ask any questions about the research including issues about confidentiality, the findings of the research and where the research will be disseminated before being asked to agree to each item on the consent form.*
- *Start audio-recording*
- *Go through each item on the consent form and record their verbal consent. Explain that you will send/email a copy of the consent form for their records. They should already have a copy of the participant information sheet with details about the study, how to withdraw etc*
- *Explain that they don’t have to answer all the questions just because they have consented to the interview, and that they can take a break or stop the interview at any time.*
- *Explain that you are there to understand more about their experiences and that they will have some time at the end of the interview to talk about any other issues that are important to them that may not have been covered by the questions.*
- *Check that they are happy to continue to be audio-recorded.*
- *Begin the interview.*

**TOPICS TO BE COVERED IN THE INTERVIEW**

Patient’s reflections on living with/managing long-term conditions

- Could you tell me about your health conditions? Prompts – how long have you had conditions, how have they developed over time
- What are your experiences of managing your long-term conditions? Prompts – medication, self-management, navigating primary/specialist care, relationships with clinicians/HCPs

Understanding of shared decision-making

- What do you understand by the idea of making shared decisions about your care and/or medications with your doctor or other clinician (shared decision-making)?
- What does it mean to you?

Experiences of shared decision-making

- What has been your role in decisions made about your care/ medications?
- How do decisions get taken on management of your condition (e.g. medications), does your doctor mainly decide, is the decision shared or do you mainly decide?
- If they feel they have no role: Does your doctor ask your opinion about starting a new treatment? Are there things you have asked your doctor to take into account (e.g., possible side effects that are important to avoid for you/ ways to take the treatment/ time of day to take the treatment?)
- What are your experiences of shared decision-making? Prompt – think through a typical consultation – how do you work with your doctor to reach decisions about your care and medication?
- What challenges are there in shared decision-making for you?
- Has your doctor ever used any information as part of shared decision-making? (e.g. information from a leaflet/ poster/ print-out from a computer?)
  - How? / What information?
- Does your doctor use information from their computer as part of shared decision-making?
  - How did they use the computer and screen?
  - Did they move their screen so that you could see the information on their computer screen? How?
  - Do you sometimes look up information on a computer before or after you have met with your doctor? What kinds of information? How do you use this information in a consultation?

Prior use of AI or risk assessment tools / information in shared decision making

- Brief check about their understanding about AI in general terms (briefing guide of discussion points about AI).
- Do you have any experience of a doctor using prompts on their computer (e.g. warnings about medications) as part of shared decision making/ your consultation?
- Do you have any experience of your doctor using information about risks of side-effects or harmful outcomes in shared decision-making/ as part of your consultation?
- Do you have any experience of a doctor using any other information on risks or recommendations on medication as part of shared decision-making/ as part of your consultation? What kind of information and how was it used? Does your doctor ever talk about how different medications might interact with one another? Does your existing treatment ever affect decisions about new treatment choices?

Preferences for types of information on risk presented for shared decision-making.

**Thinking about a typical consultation with your doctor and making shared decisions about management of your conditions and medication**.

These are the kinds of information about risk and medication that the AI tool will create for use within a consultation [Show and explain mock-up of information generated by automated risk assessment. Make it clear that the conditions may not be the same as their conditions, but something similar would be generated that is designed specifically for them. Explain any terms or conditions they are not clear about].

- How would you feel about you and your doctor using this information to inform shared decision-making/ as part of your consultation?
- How would you use the information? How would you expect your doctor to use it?
- Would it matter to you where the information had come from? Some information might be from data generated by a large number of patient records rather than from a randomised control trial (RCT) – would this be important to you?
- What would influence how comfortable you would feel with this information being used?
- Would the type of the information affect how comfortable you would feel? Why?
- Are there parts that you would find more or less relevant?
- What would make you more/ less comfortable with the information being used?
- How would it change your consultation with your doctor?
- Are there examples where you would be more/ less comfortable with you and your doctor using the tool?
- What kind of things would influence how you feel?
- Would you want your doctor to show you all this information?
  - Why?
  - What challenges might there be?
- Would you want to be able to look at this information (at home) before or after your consultation?
  - Why?
  - What challenges might there be?
  - How would you look at this information (on a phone, PC, tablet etc.)?
  - How would you use this information as part of your consultation?
- Do you think that using the information would change your decisions? Why? Is there any information you would want to know that isn't included in this example?

Preferences for look and feel for how the information is presented

We will present to the participant two contrasting examples in relation to the kinds of risks the tool will generate and different ways of presenting the information.

*Show first example*

**Imagine the AI tool produces this visual information while you are in your consultation with your doctor.**

- Do you think that you would feel comfortable with this visual information being used as part of your consultation? Why?

***If not*** – ask

- What might be more useful for you?

***If yes*** – probe for

- How would you use the visual information?
- Which part of the visual information is most helpful? Why?
- Which parts are less helpful?
- What other information would be helpful?
- Do you have any preferences for how the information should be presented? Look and feel
- Would you want your doctor to show it to you?
  - Why?
  - What challenges might there be?
- Do you think that using the visual information would change the consultation? Why?

*Show second example*

*Repeat questions*

Conclusion of interview

Now we’ve talked a bit about how the outputs from the AI tool could be used in your consultation, how do you feel about it?

Thinking back to the difficulties you talked about in managing treatments, do you think the visual information from the AI tool could have an effect on this?

Thank you. That was my last question.

Is there anything you would like to add about the things we talked about but have not covered in the interview?

*Any questions from interviewee*

*Reminder of study contact details and signposting*
